# Essential Oil of *Calotropis procera*: Comparative Chemical Profiles, Antimicrobial Activity, and Allelopathic Potential on Weeds

**DOI:** 10.3390/molecules25215203

**Published:** 2020-11-09

**Authors:** Saud L. Al-Rowaily, Ahmed M. Abd-ElGawad, Abdulaziz M. Assaeed, Abdelbaset M. Elgamal, Abd El-Nasser G. El Gendy, Tarik A. Mohamed, Basharat A. Dar, Tahia K. Mohamed, Abdelsamed I. Elshamy

**Affiliations:** 1 Plant Production Department, College of Food & Agriculture Sciences, King Saud University, P.O. Box 2460, Riyadh 11451, Saudi Arabia; srowaily@ksu.edu.sa (S.L.A.-R.); assaeed@ksu.edu.sa (A.M.A.); baseratali@gmail.com (B.A.D.); 2 Department of Botany, Faculty of Science, Mansoura University, Mansoura 35516, Egypt; 3 Department of Chemistry of Microbial and Natural Products, 33 El-Bohouth St., Dokki, Giza 12622, Egypt; algamalgene@yahoo.com; 4 Medicinal and Aromatic Plants Research Department, National Research Centre, 33 El Bohouth St., Dokki, Giza 12622, Egypt; aggundy_5@yahoo.com; 5 Chemistry of Medicinal Plants Department, National Research Centre, 33 El-Bohouth St., Dokki, Giza 12622, Egypt; tk.mohamed@nrc.sci.eg; 6 Department of Natural Compounds Chemistry, National Research Centre, 33 El Bohouth St., Dokki, Giza 12622, Egypt; ta.mourad@nrc.sci.eg

**Keywords:** Sodom’s apple, volatile organic compounds, terpenes, phytotoxicity, biological activity

## Abstract

Plants are considered green resources for thousands of bioactive compounds. Essential oils (EOs) are an important class of secondary compounds with various biological activities, including allelopathic and antimicrobial activities. Herein, the present study aimed to compare the chemical profiles of the EOs of the widely distributed medicinal plant *Calotropis procera* collected from Saudi Arabia and Egypt. In addition, this study also aimed to assess their allelopathic and antimicrobial activities. The EOs from Egyptian and Saudi ecospecies were extracted by hydrodistillation and analyzed via GC-MS. The correlation between the analyzed EOs and those published from Egypt, India, and Nigeria was assessed by principal component analysis (PCA) and agglomerative hierarchical clustering (AHC). The allelopathic activity of the extracted EOs was tested against two weeds (*Bidens pilosa* and *Dactyloctenium aegyptium*). Moreover, the EOs were tested for antimicrobial activity against seven bacterial and two fungal strains. Ninety compounds were identified from both ecospecies, where 76 compounds were recorded in Saudi ecospecies and 33 in the Egyptian one. Terpenes were recorded as the main components along with hydrocarbons, aromatics, and carotenoids. The sesquiterpenes (54.07%) were the most abundant component of EO of the Saudi sample, while the diterpenes (44.82%) represented the mains of the Egyptian one. Hinesol (13.50%), *trans*-chrysanthenyl acetate (12.33%), 1,4-*trans*-1,7-*cis*-acorenone (7.62%), phytol (8.73%), and myristicin (6.13%) were found as the major constituents of EO of the Saudi sample, while phytol (38.02%), *n*-docosane (6.86%), linoleic acid (6.36%), *n*-pentacosane (6.31%), and bicyclogermacrene (4.37%) represented the main compounds of the Egyptian one. It was evident that the EOs of both ecospecies had potent phytotoxic activity against the two tested weeds, while the EO of the Egyptian ecospecies was more effective, particularly on the weed *D. aegyptium*. Moreover, the EOs showed substantial antibacterial and antifungal activities. The present study revealed that the EOs of Egyptian and Saudi ecospecies were different in quality and quantity, which could be attributed to the variant environmental and climatic conditions. The EOs of both ecospecies showed significant allelopathic and antimicrobial activity; therefore, these EOs could be considered as potential green eco-friendly resources for weed and microbe control, considering that this plant is widely grown in arid habitats.

## 1. Introduction

Ever since ancient times, humans have depended on plants as sources of food and medicine [1]. Nowadays, plants are integrated as green resources of safe bioactive materials [2,3]. Xerophytes (desert plants) are considered rich in bioactive secondary metabolites, where they metabolize these bioactive compounds as a defense strategy [4,5,6,7,8]. The medicinal functions of the natural products and secondary metabolites of wild plants are confirmed as inhibitors of many diseases and infections, as well as antioxidant, antimicrobial, anti-inflammatory, and phytotoxicity [9,10,11,12]. Among the bioactive compounds, essential oils (EOs) are the characteristic components of almost all the aromatic and medicinal plants with several bioactivities [10,13,14,15].

Several factors have been reported to influence the chemical composition of the EO such as habitat, salinity, temperature, altitude, seasonality, plant age and development, and water availability [9,11,16,17]. In consequence, the bioactivity of the EO is affected by the factors mentioned above. The EO analysis of different organs of *Lantana camara* showed variations regarding not only organ but also the collection period [18]. 

*Calotropis procera* (Aiton) W.T. Aiton (Family Apocynaceae) is known as Sodom’s apple. It is a widely distributed plant worldwide, particularly in arid and semi-arid areas. It is a native tree to northern Africa, the Arabian Peninsula, the Middle East, and southern Asia. Additionally, it is considered a weed in several habitats such as roadsides, waste areas, near watercourses, disturbed sites, open woodlands, sand dunes, grasslands, and pastures [19]. *Calotropis procera* is used excessively in folk medicines for the treatment of cold, fever, leprosy, asthma, rheumatism, eczema, indigestion, diarrhea, elephantiasis, skin diseases, and dysentery [20,21]. Several pharmacological activities were documented for different extracts of *C. procera* such as anticancer, anti-inflammatory, antidiabetic, gastroprotective, cardiovascular, antipyretic, antioxidant, anthelmintic, anti-angiogenic, hypolipidemic, antimicrobial, analgesic, and anticonvulsant [7,22,23,24,25,26]. Due to the high biological impacts of this plant, many studies identified the presence of several metabolites such as flavonoids, tannins, terpenoids, saponins, alkaloids, steroids, and cardenolides [24,26,27]. Additionally, the EO of this plant was studied for Indian [28], Nigerian [29,30], and Egyptian ecospecies [31]. However, no study explored the variation of the EO of this tree with different climatic and environmental conditions. Many studies have proposed that the chemical composition either in quality or quantity is substantially affected by the variation in the climatic and environmental conditions [11,14,32,33]. Therefore, the present work aimed to, (i) study the comparative chemical profiles of the EOs of *C. procera* collected samples from Saudi Arabia and Egypt, as well as with the other reported ecospecies, (ii) assess the phytotoxic effects of EOs of both samples on the weeds *Bidens pilosa* L. and *Dactyloctenium aegyptium* (L.) Willd., and (iii) evaluate antimicrobial activities of EOs against some bacterial and fungal strains. 

## 2. Results and Discussion

### 2.1. Chemical Profiles of EOs of C. Procera 

The EOs extracted hydrodistillation from both Saudi and Egyptian ecospecies of *C. procera* collected are yellow-colored with a quantity of 0.046% and 0.029% (*v*/*w*), respectively. Quantity variation of the EOs between the two plant samples might be attributed to the effects of environmental factors such as habitat, salinity, temperature, altitude, seasonality, plant age and development, and water availability [9,11]. The extracted EOs were analyzed via GC-MS, where 86 chemical compounds, were characterized in the two *C. procera* samples (Table 1). From these compounds, 76 were detected in the Saudi sample while 33 were recorded in the Egyptian sample. In addition to the variations in the yield of the oil of the two plant samples, the GC-MS exhibited substantial qualitative variation, whereas the Saudi sample was very rich in compounds compared to the Egyptian one. This significant variation could be attributed to the variation in habitat, temperature, altitude, and soil conditions [11,16].

**Figure 1:**
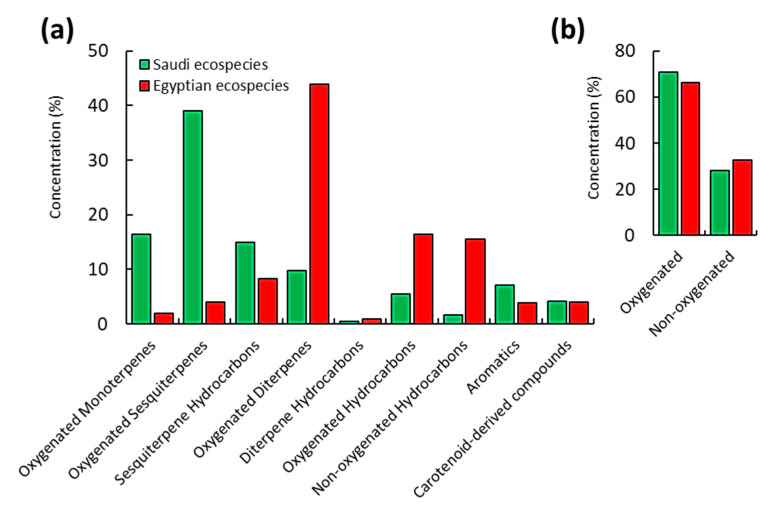
Concentrations of the various classes of the identified compounds. (**a**) the nine identified classes and (**b**) the oxygenated and non-oxygenated components.

The identified compounds can be categorized under nine classes. The oxygenated compounds were the most represented, where they attained 70.83% and 66.22% of the Saudi and Egyptian ecospecies, respectively (Figure 1). Oxygenated sesquiterpenes were the major class in the Saudi samples (39.10%), while oxygenated diterpenes represent the major class in the Egyptian sample (43.92%).

Chemical profiling of the two EOs exhibited the abundance of terpenoid compounds, including mono-, sesqui-, and diterpenes, as main components, in addition to hydrocarbons, aromatics, and carotenoid-derived compounds. The chemical characterization of EO components of Saudi *C. procera* via GC-MS analysis revealed the abundance of sesquiterpenes (54.07%) that categorized to oxygenated sesquiterpenes (39.10%) and non-oxygenated sesquiterpenes (14.97%). Hinesol (13.50%) and 1,4-*trans*-1,7-*cis*-acorenone (7.62%) were identified as the major oxygenated sesquiterpenes. On the other side, (*E*,*E*)-farnesyl acetone represented the minor one with a concentration of 0.16% (Figure 1, Table 1). Among all the sesquiterpene hydrocarbons, *α*-cadinene (3.31%) and *α*-cubebene (2.19%) represented the main constituents, while *α*-muurolene (0.15%) was the minor one.

Sesquiterpenes were present in a remarkable concentration in the EO of Egyptian ecospecies, with a total concentration of 12.92%, which is divided into oxygenated and non-oxygenated compounds (4.02% and 8.27%, respectively). The oxygenated sesquiterpenes are comprised of seven compounds. Viridiflorol (2.47%) and (8*S*,14)-cedrandiol (1.38%) were the main compounds, while *α*-acorenol was detected as minor. Among all the identified sesquiterpenes, only three non-oxygenated compounds, *trans*-caryophyllene (3.07%), *cis*-thujopsene (0.83%), and bicyclogermacrene (4.37%), were identified.

Sesquiterpenes represent one of the principal components of terpenes of EOs and were basically established biosynthetically via the pathways of isoprenoid [34]. In the plant kingdom, sesquiterpenes were found as main components of EOs from many plants such as *Lactuca serriola* [35], some *Launaea* species [11], *Eucalyptus camaldulensis* [36], *Teucrium maghrebinum* [37], some *Salvia* species [38], and others. In the present study, the EO of the Saudi plant exhibited a clear difference with the previously published profiles of different organs of Egyptian *C. procera* [31] as well as Indian *C. gigantea* [28]. However, the Egyptian ecospecies exhibited consistency in the EO chemical composition, with these published data, where sesquiterpenes were identified as main constituents.

The monoterpenes in EO of Saudi *C. procera* represented remarkable concentration (16.48%). The oxygenated monoterpenes, *trans*-chrysanthenyl acetate (12.33%), and camphor (1.50%) were found as the main compounds, while *α*-cyclocitral (0.15%) was detected as a minor compound (Table 1). In contrast to the Saudi plant, the monoterpene was found as minor constituents of EO from the Egyptian plant, comprising only three identified oxygenated monoterpenes. Among all the identified oxygenated monoterpenes, *trans*-chrysanthenyl acetate (1.09%) was found as the major compound, while eucalyptol (0.21%) represented a minor one. Monoterpenes were found as main components of EOs derived from several aromatic plants and herbs such as *Euphorbia heterophylla* [39], *Salvia sclarea* [40], *Callistemon viminalis* [41], *Thymus eigii* [42], and others. Regarding *Calotropis* species, monoterpenes were characterized as main compounds in EOs derived from Indian *C. gigantea* [28] that disagree with present results. The present results were completely in agreement with the described chemical profile of EO of stems, leaves, flowers, and fruits of *C. procera* collected from the Egyptian delta [31], in which the monoterpenes represented low concentrations.

Diterpenes were found in a remarkable concentration (10.17%) of the Saudi ecospecies. Four diterpenoids were characterized, including three oxygenated diterpenes, phytol (8.73%), isophytol (0.25%), and *trans*-geranyl geraniol (0.80%), as well as one diterpene hydrocarbon (kaur-16-ene).

In contrast, the EO of Egyptian plant was found to contain 44.82% of diterpenes out of the overall constituents with an abundance of oxygenated diterpenes (43.92%) in addition to traces of diterpene hydrocarbons (0.90%). The oxygenated diterpenes were represented by only three compounds, phytol (38.02%), isophytol (2.66%), and *trans*-geranyl geraniol (3.24%). The plants that are characterized by the preponderance of diterpenes are very rare in the plant kingdom [39,43]. These results were in harmony with that described for different organs of Egyptian *C. procera*, in which the diterpenes were reported as main compounds, particularly *E*-phytol [31]. Nevertheless, the present data were inconsistent with the profile of Indian *C. gigantea* [28], in which the diterpenes were completely absent. From the EO chemical profile of the Egyptian *C. procera*, it can be concluded that this plant is a very specific plant due to its ability to biosynthesize diterpenes, especially phytol.

Out of the total identified mass of the Saudi sample, the oxygenated hydrocarbon represented by 5.47%, while the non-oxygenated hydrocarbon attained 1.60%. Methyl palmitate (2.86%) was found as major oxygenated hydrocarbons, while linoleic acid was characterized as a minor one. On the contrary, the hydrocarbons represented the second class rank in the EO of the Egyptian sample with a concentration of 31.96%. Oxygenated hydrocarbons (16.37%) were represented with six compounds, in which linoleic acid (6.36%) and oleic acid (3.51%) were found as main compounds. The non-oxygenated hydrocarbons attained 15.59%, including *n*-docosane (6.86%) and *n*-pentacosane (6.31%) as majors as well as *n*-nonadecane (0.45%) as minor (Table 1).

Low concentrations of aromatic compounds were identified, with a concentration of 7.00% and 3.82% from the Saudi and Egyptian samples, respectively. Seven aromatic compounds were characterized in EO of the Saudi sample, including myristicin (6.13%) as the main constituent and bolandiol (0.17%) as minor. The profile of the Egyptian plant revealed that myristicin (2.09%) and ethyl iso-allocholate (1.58%) were major components, while bolandiol (0.15%) was minor. From the previously described chemical profiles and the reported data, myristicin was found as a characteristic compound in *Calotropis* species [28,29,31]. Myristicin was found with a concentration of 26.4%, 28.1%, 27.9%, and 25.9% of the leaves, stems, flowers, and fruits of *C. procera* collected from Egypt, respectively. Moreover, the leaves of Nigerian *C. procera* were deduced to have myristicin as the main compound with 19.9% of the total mass [29].

In the present study, carotenoids were determined in Saudi ecospecies, comprising five compounds *α*-ionone and its two derivatives in addition to *α*-damascenone and dihydroedulan II. On the other hand, three carotenoids were characterized in the Egyptian sample, including *trans*-*α*-ionone (2.91%) and *α*-iso methyl ionone (0.38%) as well as *α*-damascenone (0.71%). Overall, the determined significant difference in chemical components between Saudi and Egyptian *C. procera* might be ascribed to the variation of biosynthesis of the metabolites, including EOs. The biosynthesis of EOs components is directly affected by environmental and climatic factors such as temperature, altitude, seasonality, plant age, and water availability as well as the soil conditions [16,33,44].

### 2.2. Principal Components Analysis (PCA) and Agglomerative Hierarchical Clustering (AHC)

**Figure 2:**
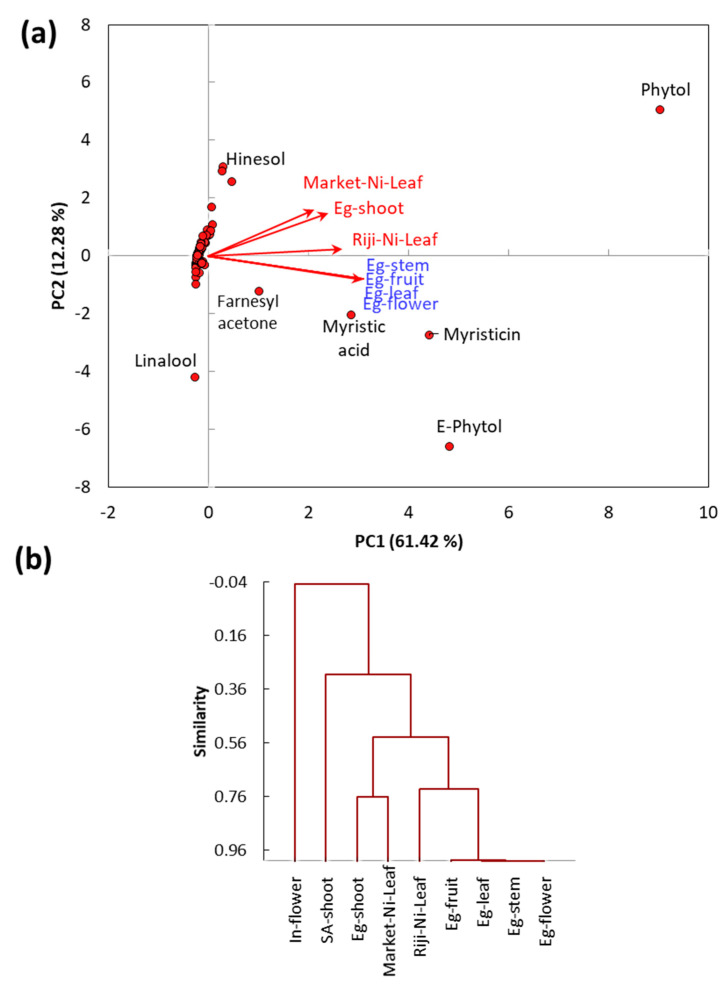
(**a**) Principal component analysis (PCA) and (**b**) agglomerative hierarchical clustering (AHC) based on the chemical composition of the EO derived from shoots of both Egyptian and Saudi ecospecies of *C. procera* as well as the reported EO from Nigerian (Ni), Indian (In), and Egyptian (Eg) ecospecies.

To determine the correlation among either the studied *Calotropis* ecospecies (Saudi and Egyptian) in the present study or in the other reported ecotypes (Indian [28], Nigerian [29,30], and Egyptian [31]), we subjected the EO chemical compounds to PCA and AHC. The PC1 axis showed 61.42% of the total variance, while the PC2 axis a further 12.28% (Figure 2a). It is clear that the reported Egyptian ecotype by Wahba and Khalid [31] was separated from the other ecotypes, whereas the EOs from all organs (stem, leaf, fruit, and flower) were similar in the chemical composition. Furthermore, the EOs from these Egyptian samples were characterized by farnesyl acetone, myristic acid, and myristicin (Figure 2a).

On the other side, the Egyptian ecotype of the present study showed a close correlation to the Nigerian leaf samples collected from the market, and these samples are characterized by the high content of phytol and hinesol. However, the sample collected from Riji region of Nigeria showed more variation than those collected from the market (Figure 2a).

The AHC analysis showed that the examined samples can be clustered into 5 groups: (1) Egyptian samples of the stem, leaf, fruit, and flower collected from Nasr City, (2) Nigerian leaf sample collected from Riji, (3) present Egyptian samples and Nigerian leaf sample collected from the market, (4) Saudi sample, and (5) Indian sample (Figure 2b). The variation among different samples could be ascribed to the variation in the climatic and edaphic conditions as well as the difference in the genetic pool [32,45].

### 2.3. Allelopathic Activity of the EOs

**Figure 3:**
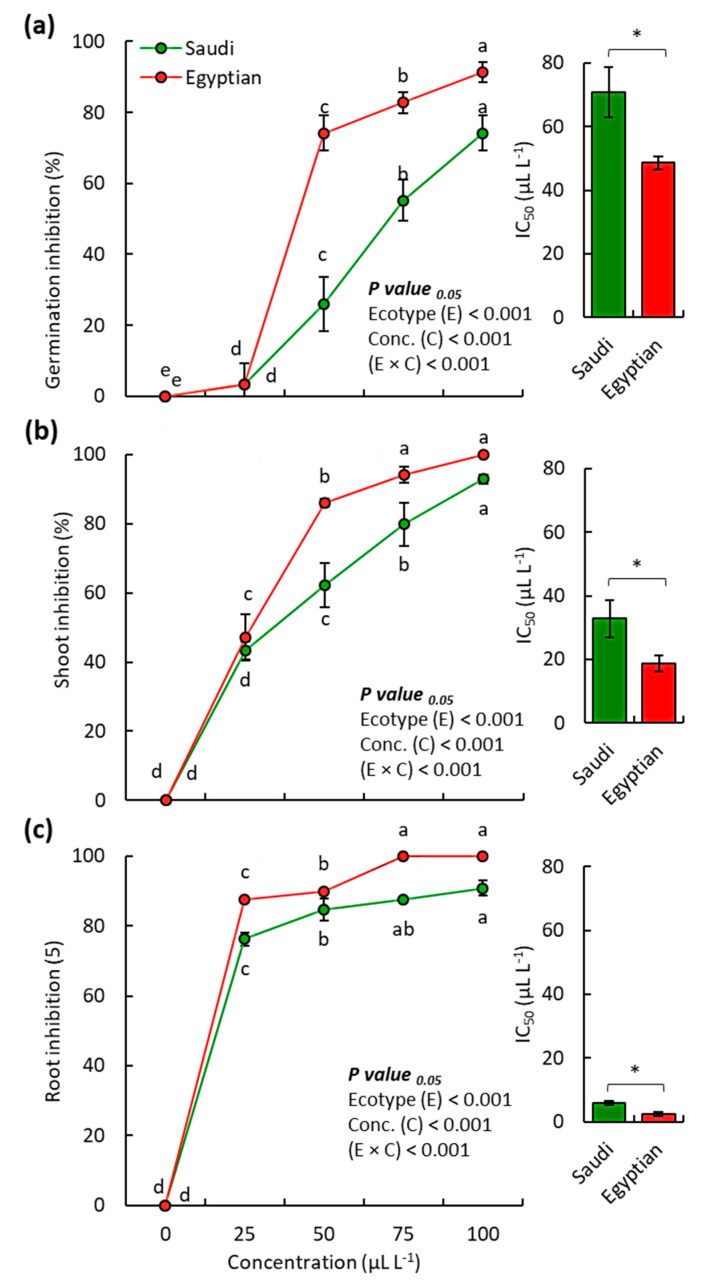
Phytotoxic activity of the EO from Saudi and Egyptian ecotypes of *C. procera* on the (**a**) seed germination, (**b**) seedling shoot growth, and (**c**) seedling root growth of *Bidens pilosa*. * *p* < 0.05 (two-tailed *t*-test). Different letters per each line mean significant difference (one-way randomized blocks ANOVA). Data are mean value (*n* = 3) and the bars represent the standard error.

The EOs from both Egyptian and Saudi ecospecies exhibited significant allelopathic activity (*p* < 0.05) against the weed *B. pilosa* (Figure 3, Table S1). The germination of *B. pilosa* was reduced by 91.7% and 75.0% when treated with 100 µL L^−1^ of the EO from Egyptian and Saudi ecospecies of *C. procera*, respectively (Figure 3a).

On the other hand, the shoot growth and root growth of *B. pilosa* seedlings were more affected than the germination, where the root growth was inhibited by 100.0% and 92.9%, for Egyptian and Saudi ecospecies, respectively (Figure 3b). The seedling shoot growth showed a 100.0% and 90.9% reduction under the same treatment (Figure 3a). It was clear that the root was affected by the EOs more than shoot, which could be ascribed to the direct contact with the EOs or due to the permeability of the membrane of root cells [14,32,39].

Based on the IC_50_ values, it was observed that the EO of Egyptian ecospecies showed more phytotoxic activity on *B. pilosa* than Saudi ecospecies. The Egyptian ecospecies attained an IC_50_ value of 48.6, 18.7, and 2.3 µL L^−1^ for germination, seedling shoot growth, and seedling root growth, respectively, while the Saudi ecospecies showed an IC_50_ value of 70.9, 32.9, and 5.9 µL L^−1^, respectively (Figure 3). The potency of the EO from Egyptian ecospecies on the germination, seedling shoot growth, and seedling root growth of *B. pilosa* were ≈ 1.5–1.8, and 2.5-fold of Saudi ecospecies.

The weed *B. pilosa* has been reported as a noxious weed in many countries and infests many crops. It is characterized by efficient seeds dispersion [46]. The allelopathic control of the EOs from various plants was tested against this weed such as *Cullen plicata* [13], *Xanthium Strumarium* [32], and *Tagetes minuta* [47]. The EO from *C. plicata* showed comparable phytotoxic activity on the germination of *B. pilosa*, where it attained IC_50_ values of 49.4 µL L^−1^ [13]. The EO of *C. procera* in the present study revealed stronger activity than those of *C. plicata* on radicle growth and shoot growth of *B. pilosa*.

**Figure 4:**
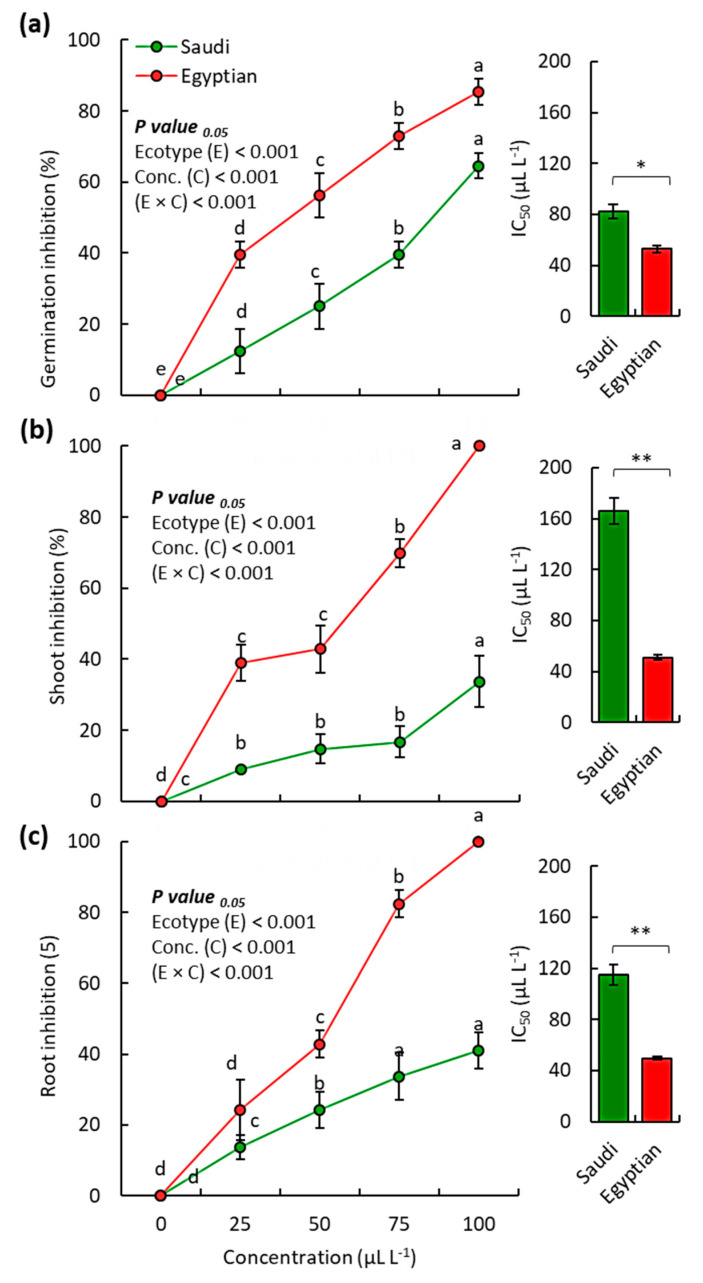
Phytotoxic activity of the EO from Saudi and Egyptian ecotypes of *C. procera* on the (**a**) seed germination, (**b**) seedling shoot growth, and (**c**) seedling root growth of *D. aegyptium*. * *p* < 0.05, ** *p* < 0.01 (two-tailed *t*-test). Different letters per each line mean significant difference (one-way randomized blocks ANOVA). Data are mean value (*n* = 3) and the bars represent the standard error.

In addition, the EOs of both Egyptian and Saudi ecospecies showed also substantial phytotoxic activity against the seed germination and seedling growth of the weed *D. aegyptium* (Figure 4). At the highest concentration (100 µL L^−1^) of the EO from Egyptian ecospecies, the germination, shoot growth, and root growth of *D. aegyptium* declined by 85.4%, 100%, and 100%, respectively, while the EO of Saudi ecospecies showed 64.6%, 33.7%, and 41.1%, respectively (Figure 4).

Based on the IC_50_ data, it was evident that EO from Egyptian ecospecies was more effective against the weed *D. aegyptium* since the Egyptian sample had IC_50_ values of 52.4, 51.1, and 49.5 µL L^−1^, for the germination, seedling shoot growth, seedling root growth, respectively, while the Saudi sample attained IC_50_ values of 82.5, 166.0, and 114.9 µL L^−1^, respectively. The influence of the EO from Egyptian ecospecies on the germination, seedling shoot growth, and seedling root growth of *D. aegyptium* was ≈1.6, 3.2-, and 2.4-fold of Saudi ecospecies.

Overall, it was evident that the EOs of both ecospecies had potent phytotoxic activity against the two tested weeds (*B. pilosa* and *D. aegyptium*), while the EO of the Egyptian ecospecies was more effective, particularly on the weed *D. aegyptium*. Consistent with the present results, the EO from aboveground parts of *Pulicaria somalensis* has been reported to have a stronger allelopathic effect on *D. aegyptium* than *B. pilosa* [10]. This could be attributed to the genetic resistance of weeds [48].

The inhibitory activity of the EOs from Egyptian *C. procera* in the present study could be ascribed to the presence of oxygenated terpenoid compounds, particularly the major compounds such as phytol, *n*-docosane, linoleic acid, *n*-pentacosane, and bicyclogermacrene. However, the phytotoxicity of the Saudi *C. procera* could be attributed to the content of major constituents such as hinesol, *trans*-chrysanthenyl acetate, 1,4-*trans*-1,7-*cis*-acorenone, phytol, and myristicin. The hinesol has been reported as an antitumor agent [49] and an anti-gastric ulcer agent [50]. However, no study dealt with the allelopathic/phytotoxic activity of this compound; thereby, further study is recommended to determine the allelopathic activity of this compound in a pure form and to assess its mode of action(s) and its biosafety as a bioherbicide. The EOs rich with *trans*-chrysanthenyl acetate showed potential allelopathic activity [34,51,52]. The diterpene was considered as rare compounds in plants, while an oxygenated diterpenes phytol was reported here as a major compound in both Egyptian and Saudi ecospecies of *C. procera*. The EO of *Euphorbia heterophylla* has been reported as rich with phytol, whereas this EO exhibited an allelopathic activity on *Cenchrus echinatus* [39].

Most of these major compounds are oxygenated compounds. The oxygenated terpenes have been reported as more bioactive compounds than non-oxygenated ones due to the reactivity of the hydroxyl group [9,10,34,39]. In the present study, the oxygenated compounds represent 66.22% of the total identified compounds in the Egyptian ecospecies, while they represent 70.83% of the Saudi ecospecies.

### 2.4. Antimicrobial Activities

The EOs of *C. procera* collected from Saudi Arabia and Egypt exhibited significant antimicrobial potential against all the tested microbes, including bacteria (*Staphylococcus aureus*, *S. pyogenes*, *S. epidermidis*, *Salmonella typhi*, *Escherichia coli*, *Shigella* spp., and *Pseudomonas aeruginosa*) and fungi (*Trichophyton shoenlenii* and *Aspergillus fumigatus*), in a concentration-dependent manner (Table 2). The ANOVA test revealed a significant difference between the two ecospecies, except in the case of *S. pyogenes* and *T. shoenlenii* (Table S2).

The EO of the Saudi plant exhibited antibacterial activity against all the positive and negative bacterial strains with an inhibition zone ranging from 5.60 to 37.00 mm. At the highest concentration (2.50 µg mL^−1^), *S. typhi* was most sensitive, while *E. coli* showed the lowest sensitivity. The results of the EO activity against fungi revealed that *T. shoenlenii* is more sensitive than *A. fumigatus* (Table 2).

The minimum inhibitory concentration (MIC) data revealed that it ranged from 0.16 to 0.23 µg mL^−1^, where the bacterial strains can be arranged according to the sensitivity as the following sequence: *S. aureus* > *S. epidermidis* > *S. typhi* > *S. pyogenes* > *E. coli > P. aeruginosa*. The EO of *Paramignya trimera* showed similar inhibition on a set of microbes and, interestingly, the activity revealed the comparable sequence, where *S. aureus* affected more than *E. coli* and *P. aeruginosa* [53]. Additionally, the EO of this plant exhibited MIC values 21.00 and 28.00 µg mL^−1^ against *T. shoenlenii* and *A. fumigatus*, respectively (Table 2).

On the other hand, the EO of the Egyptian ecospecies showed significant antibacterial activity against the tested bacterial strains, where it attained an inhibition zone ranging from 8.50 to 35 mm. At the highest concentration (2.50 µg mL^−1^), *Shigella* spp. was the most inhibited strain and *E. coli* was the lowest inhibited one (Table 2). Additionally, the EO of the Egyptian ecospecies exhibited maximum antifungal activity against *A. fumigatus*, while it attained an inhibition zone of 31.30 mm against *T. shoenlenii*. Based on the MIC data, it is clear that the EO of Egyptian ecospecies has comparable activity against *S. aureus*, *S. pyogenes*, *S. epidermidis*, *S. typhi*, *E. coli*, and *Shigella* spp., while *P. aeruginosa* was more resistant (Table 2). Moreover, this EO exhibited significant antifungal activity against the two used strains of fungi, *T. shoenlenii* and *A. fumigatus*, with MIC values of 15.75 and 15.79 µg mL^−1^, respectively (Table 2).

Generally, the Egyptian ecospecies showed more antimicrobial activity compared to the Saudi ecospecies, although the Egyptian ecospecies have a lower number of compounds. This difference could be ascribed to the variation in the chemical composition of the EO. The bioactivities of the EOs are directly correlated with their chemical compound compositions [14,43]. The chemical compounds of the EOs contribute to the bioactivity either individually or synergistically [13,16]. The terpenoid compounds represented the main constituents of the EOs derived from Saudi and Egyptian *C. procera* (Table 1). Total terpenes, including mono, sesqui, and diterpenes, have been reported to possess an essential role in the growth inhibition of many microbes [43,54,55]. Due to the reactivity of the hydroxyl groups, the highly oxygenated compounds in the EOs lead to more antimicrobial activity [43,56]. In this context, the oxygenated terpenes in the EO of Saudi ecospecies represented 65.39% of the total mass, in addition to 13.53% of other oxygenated compounds, such as hydrocarbons and aromatics. This high content of the oxygenated compounds could be attributed to the observed antimicrobial activity of *C. procera* EO. Many studies proved that sesquiterpenes are potent antimicrobial agents [54,57,58,59].

In the EO of Saudi ecospecies, the major compounds (hinesol, *trans*-chrysanthenyl acetate, 1,4-*trans*-1,7-*cis*-acorenone, phytol, and myristicin) were described to have a significant antimicrobial role of EOs of several plants such as *Cyclotrichium leucotrichum* [60], *Tanacetum santolinoides* [61], *Bupleurum plantagineum* [62], and *Daucus littoralis* [63]. On the other side, the major compounds in the EO of Egyptian ecospecies (phytol, *n*-docosane, linoleic acid, *n*-pentacosane, and bicyclogermacrene as major constituents) have been reported as major constituents of other reported EO with antimicrobial activity from various plants such as *Tamarix boveana* [64], *Laurus nobilis*, *Prunus armeniaca* [65], and *Ferula szovitsiana* [66]. Phytol, the major diterpenoid in EOs of both plant samples, was stated as a common compound in EOs with a potential role as an antimicrobial agent [67].

Overall, our findings revealed the potent antimicrobial activity of EOs from *C. procera*. Thereby, these EOs may be considered as promising natural eco-friendly agents for antimicrobial drugs. Furthermore, the antifungal activity of the present EO revealed that this oil could be used as a food preservative [68]. Nevertheless, further study is recommended to evaluate the activity of the major compounds in pure form, either singular or in combination, particularly hinesol and *trans*-chrysanthenyl acetate.

## 3. Materials and Methods

### 3.1. Plant Materials Collection and Identification

**Figure 5:**
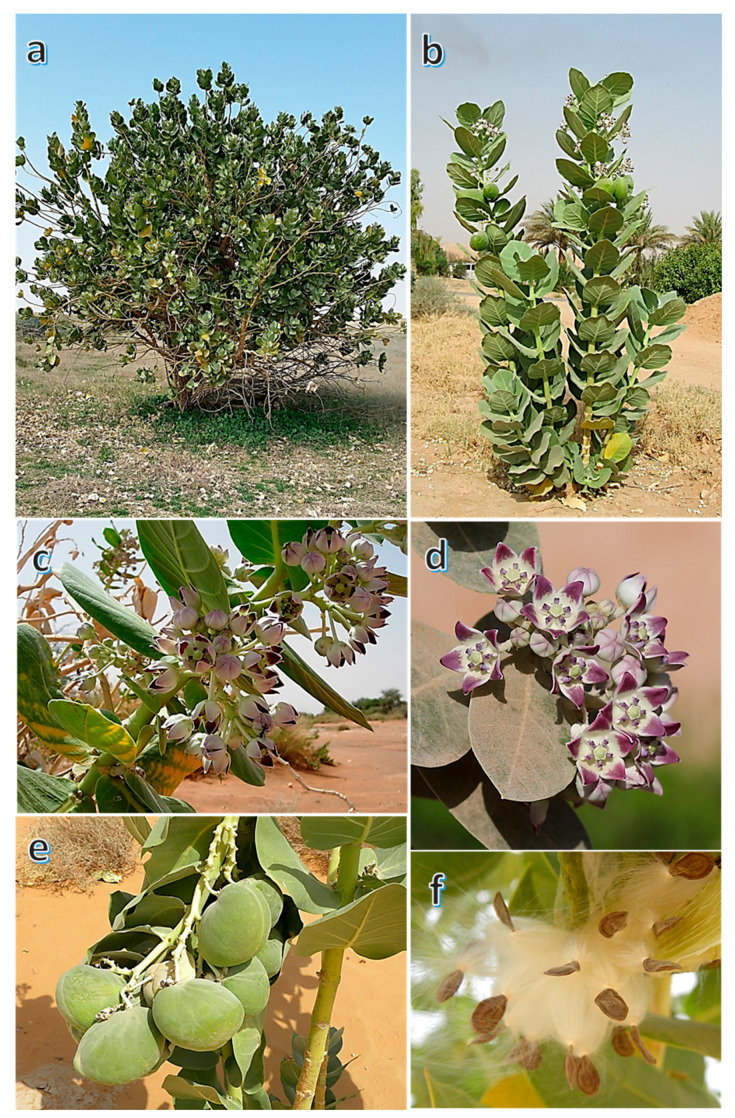
*Calotropis procera* (Aiton) W.T. Aiton (**a**) Overview of an old tree, (**b**) Overview of a young tree, (**c**) Close view of flowering branch, (**d**) flowers, (**e**) fruits, and (**f**) seeds.

The fresh and healthy branches of *C. procera* were collected from Raudhat Khuraim, located about 100 km away from Riyadh, Saudi Arabia (25°23′28.1″ N 47°15′44.1″ E), and another sample was collected from Wadi Hagul, northwest Suez Gulf, Egypt (29°54′02.7″ N 32°13′05.5″ E) (Figure 5). Two samples per each ecospecies were collected in plastic bags during the spring (in March) of 2019 and transferred to the laboratory.

The Saudi specimen was identified according to Chaudhary [69] and Collenette [70], while the Egyptian plant specimen was identified according to Tackholm [71] and Boulos [72]. A voucher specimen of the collected plant is released in the herbarium of either King Saud University (code: KSU-001003016) and National Research Center (code: CP-NRC-XC 091178). The plant materials were dried in shade at room temperature (25 ± 3 °C) for two weeks (until complete dryness), ground into a fine powder, and packed in a paper bag.

### 3.2. Essential Oil Extraction, GC-MS Analysis, and Constituents’ Identification

The EOs of 200 g from the prepared plant samples were extracted by hydrodistillation from the shoots of Saudi and Egyptian *C. procera* (two samples for each) via a Clevenger-type apparatus for three hours. The apparatus has a 5000 mL round flask, filled with 2000 mL distilled water. The oil layer was collected, water was removed using 0.5 g of anhydrous Na_2_SO_4_, and stored in a dark glass vial at 4 °C till further analysis. The yields of the extracted EOs were calculated via the equation: 100 × (V/W); where V: volume of extracted EO, W: weight of the plant material used in extraction. The chemical composition of the EO samples was analyzed separately by gas chromatography-mass spectrometry (GC-MS) at the National Research Center, Giza, Egypt, as described in our previously documented work [11,32]. The device consists of TRACE GC Ultra Gas Chromatographs (THERMO Scientific™ Corporate, Waltham, MA, USA) and Thermo Scientific ISQ™ EC single quadrupole mass spectrometer. The GC-MS system is equipped with a TR-5 MS column with dimensions of 30 m × 0.32 mm internal diameter (i.d.), 0.25 µm film thickness. At a flow rate of 1.0 mL min^−1^, helium was used as carrier gas with a split ratio of 1:10. The temperature program was 60 °C for 1 min, rising by 4.0 °C min^−1^ to 240 °C and held for 1 min. A diluted sample in hexane (1 µL) at a ratio of 1:10 (*v*/*v*) was injected, and the injector and detector were held at 210 °C. Mass spectra were recorded by electron ionization (EI) at 70 eV, using a spectral range of *m*/*z* 40–450. The identification of the EO chemical components was performed via Automated Mass spectral Deconvolution and Identification (AMDIS) software, Wiley spectral library collection, NIST library database (Gaithersburg, MD, USA; Wiley, Hoboken, NJ, USA), retention indices relative to *n*-alkanes (C_8_–C_22_), or appraisal of the mass spectrum with authentic standards.

### 3.3. Allelopathic Activity of the EOs

The allelopathic activity of the EOs extracted from Egyptian and Saudi *C. procera* samples were examined against two weeds, *B. pilosa* and *D. aegyptium*. The seeds of *B. pilosa* were collected from a garden of Mansoura University, Egypt (31°02′36.5″ N 31°21′12.3″ E), while the seeds of *D. aegyptium* were collected from a field newly reclaimed near Gamasa City, northern Egypt (31°27′05.4″ N 31°27′44.2″ E). The uniform seeds were chosen, surface sterilized with sodium hypochlorite (0.3 N), and dried. The bioassay was conducted according to Abd El-Gawad [13], where serial concentrations of the EOs (25, 50, 75, and 100 µL L^−1^) were prepared using 1% Tween ^®^ 80 (Sigma-Aldrich, Darmstadt, Germany). In a Petri plate, 20 sterilized seeds were spread over a sterilized Whatman^®^ Grade 1 filter paper, and immediately 5 mL of each concentration or Tween^®^80 as a positive control. Fiver plates were performed per each treatment and the plates were sealed with Parafilm^®^ tape (Sigma, St. Louis, MO, USA) and incubated in a growth chamber at 25 ± 2 °C. After 5 and 7 days of incubation for *B. pilosa* and *D. aegyptium*, respectively, the germinated seeds were counted and the lengths of seedling roots and shoots were measured in mm. The inhibition of seed germination, seedling root, and the seedling shoot was calculated as follows:

$$\mathrm{Inhibition}\;\left(\%\right)=100\times \frac{\left(\mathrm{No}/\mathrm{Length}\;\mathrm{of}\;\mathrm{control}-\mathrm{No}/\mathrm{Length}\;\mathrm{of}\;\mathrm{tretamnet}\right)}{\mathrm{No}/\mathrm{Length}\;\mathrm{of}\;\mathrm{control}}$$

Moreover, IC_50_ was calculated graphically as the concentration of the EO required for 50% inhibition.

### 3.4. Antimicrobial Properties of EOs

The extracted EOs of the Saudi and Egyptian samples of *C. procera* were tested against some pathogenic strains of bacteria according to the technique of agar diffusion method [73], as well as fungal strains using the spore suspension method [74]. The tested bacterial strains were obtained from the American Type Culture Collection. They included either Gram-positive (*S. aureus* ATCC23235, *S. pyogenes* ATCC19615, and *S. epidermidis* ATCC12228) or Gram-negative (*S. typhi* ATCC35664, *E. coli* ATCC25922, *Shigella* spp. ATCC12040, and *P. aeruginosa* ATCC15442) bacteria, while *T. shoenlenii* ATCC22776 and *A. fumigatus* ATCC13073 were the tested fungi.

The different concentrations (2.5, 1.25, 0.625, 0.312, 0.156, and 0.078 µg mL^−1^) of EOs from Saudi and Egyptian ecospecies were mounted on a sterilized filter paper discs (∅ = 6 mm). To test the antimicrobial activity, Petri plates were inoculated with 1 × 10^6^ spores/mL of fungi (potato dextrose agar medium) and 1 × 10^8^ colony forming units (CFU)/mL of bacteria (nutrient agar medium). The discs with EO were placed in the center of the plates, and the plates were sealed with Parafilm^®^ tape (Sigma, St. Louis, MO, USA) and incubated at 37 °C for 24 h in case of bacteria and 28 °C for 72 h in case of fungi. After incubation, the diameter of inhibition zones was measured (mm) as an average of three different point measurements, as well as the minimum inhibitory concentration (MIC) was calculated. Gentamycin and amphotericin, at a concentration of 10 µg mL^−1^, were used as positive controls for antibacterial and antifungal activities, respectively.

### 3.5. Data Analysis

The data of both allelopathic and antimicrobial activities were determined in triplicates, where they are subjected to ANOVA, followed by Duncan’s test using CoStat software program (version 6.311, CoHort Software, Monterey, CA, USA). The significance of probability was adjusted at 0.05. Furthermore, the data of IC_50_ of the allopathic activity was subjected to a two-tailed *t*-test via MS Excel (2016). To assess the correlation among the studied *Calotropis* ecotypes (Egyptian and Saudi) and those reported before (Egyptian [31], Nigerian [29,30], Indian [28]), a matrix of the chemical compounds concentration percentage, derived from GC-MS analysis, was constructed. The matrix has 136 identified chemical compounds from nine samples, including (i) present Egyptian shoot, (ii) present Saudi shoot, (iii) reported Egyptian stem collected from Nasr City [31], (iv) reported Egyptian leaf [31], (v) reported Egyptian fruit [31], (vi) reported Egyptian flower [31], (vii) reported Nigerian leaf collected from Riji region [29], (viii) Nigerian leaf collected from the market [30], and Indian flower [28]. The matrix was subjected to agglomerative hierarchical cluster (AHC) and principal component analysis (PCA) with XLSTAT statistical computer software package (version 2018, Addinsoft Inc., New York, NY, USA).

## 4. Conclusions

The EOs from Egyptian and Saudi ecospecies of *C. procera* showed remarkable variation both in the number and quantity of the compound. This variation could be ascribed to the variation in the climatic, topographic, edaphic, or genetic differences. The EO of Saudi ecospecies comprised of 76 chemical compounds with hinesol, *trans*-chrysanthenyl acetate, 1,4-*trans*-1,7-*cis*-acorenone, phytol, and Myristicin as major compounds, while the Egyptian ecospecies contained 33 compounds with a dominance of phytol, *n*-docosane, linoleic acid, *n*-pentacosane, and bicyclogermacrene as major constituents. The EOs of both ecospecies had potent phytotoxic activity against the two tested weeds (*B. pilosa* and *D. aegyptium*), while the EO of the Egyptian ecospecies was more effective, particularly on the weed *D. aegyptium*. Therefore, the EO of this plant could be a promising eco-friendly bioherbicide against weeds, particularly this tree is widely grown as a weed in arid habitats. Due to the allelopathic activity of the identified compounds are still poorly understood, further study is recommended for the characterization of authentic materials of the major compounds. Moreover, evaluating their activity at the field scale, modes of action, and biosafety are required. On the other hand, the EOs showed potent antimicrobial activity that supported their leading role for antimicrobial drugs.

## Figures and Tables

**Table 1:** Chemical constituents of essential oils (EOs) of the aboveground parts of *Calotropis procera* collected from Saudi Arabia and Egypt.

No	Rt.	KI		Compound Name	Conc. (%)		Identification
		Exp	Lit		Saudi Arabia	Egypt	
**Oxygenated Monoterpenes**							
1	7.06	1030	1031	Eucalyptol	0.37 ± 0.03	0.21 ± 0.01	a & b
2	11.12	1123	1122	*α*-Cyclocitral	0.15 ± 0.02	---	a & b
3	11.45	1139	1137	*trans*-Pinocarveol	0.88 ± 0.03	---	a & b
4	12.00	1143	1143	Camphor	1.50 ± 0.06	---	a & b
5	12.75	1146	1145	Verbenol	0.33 ± 0.02	---	a & b
6	13.48	1189	1189	4-Terpineol	0.16 ± 0.02	---	a & b
7	13.61	1201	1202	Safranal	0.35 ± 0.02	---	a & b
8	15.74	1235	1234	*trans*-Chrysanthenyl acetate	12.33 ± 0.09	1.09 ± 0.04	a & b
9	16.95	1336	1338	*α*-Terpinyl propionate	0.41 ± 0.02	---	a & b
10	25.03	1453	1455	Neryl acetone	---	0.61 ± 0.03	a & b
**Oxygenated Sesquiterpenes**							
11	24.10	1499	1501	*β*-Himachalene	0.30 ± 0.01	---	a & b
12	24.32	1439	1440	*α*-Guaiene	0.17 ± 0.01	---	a & b
13	24.40	1511	1512	Germacrene D-4-ol	0.24 ± 0.02	---	a & b
14	24.90	1517	1516	6-Epishyobunol	0.28 ± 0.01	---	a & b
15	25.53	1535	1533	Nerolidol	0.64 ± 0.03	---	a & b
16	26.80	1551	1554	Diepicedrene-1-oxide	1.95 ± 0.06	---	a & b
17	27.14	1558	1557	Dihydro-*α*-agarofuran	0.86 ± 0.04	---	a & b
18	27.73	1563	1561	Hexahydrofarnesol	0.23 ± 0.02	---	a & b
19	27.95	1564	1562	Epiglobulol	0.28 ± 0.01	---	a & b
20	28.29	1567	1568	Palustrol	0.40 ± 0.03	---	a & b
21	28.52	1575	1580	Caryophyllene oxide	---	0.41 ± 0.02	a & b
22	28.77	1578	1579	Spathulenol	0.46 ± 0.02	---	a & b
23	28.94	1586	1584	Viridiflorol	2.47 ± 0.07	---	a & b
24	29.58	1588	1588	Calarene epoxide	0.21 ± 0.01	---	a & b
25	29.66	1594	1594	Isoaromadendrene epoxide	0.27 ± 0.01	---	a & b
26	29.85	1596	1597	*α*-Cedrol	0.56 ± 0.03	---	a & b
27	30.73	1604	1606	Cedrenol	0.59 ± 0.02	---	a & b
28	30.94	1619	1622	Humulane-1,6-dien-3-ol	0.78 ± 0.01	---	a & b
29	31.09	1625	1625	Aromadendrene oxide (1)	0.45 ± 0.02	---	a & b
30	31.39	1627	1628	4-epi-cubedol	1.82 ± 0.06	---	a & b
31	31.81	1632	1631	1,4-*trans*-1,7-*cis*-Acorenone	7.62 ± 0.05	---	a & b
32	32.21	1638	1638	Hinesol	13.50 ± 0.08	---	a & b
33	32.48	1649	1651	*β*-Eudesmol	0.60 ± 0.02	---	a & b
34	33.24	1657	1656	*α*-Acorenol	1.95 ± 0.05	0.35 ± 0.02	a & b
35	33.76	1669	1671	Cedr-8-en-15-ol	0.36 ± 0.02	---	a & b
36	34.23	1689	1687	Cedr-8-en-13-ol	0.78 ± 0.03	0.45 ± 0.01	a & b
37	36.89	1691	1692	Juniper camphor	0.18 ± 0.01	0.40 ± 0.02	a & b
38	38.66	1885	1885	(8*S*,14)-Cedrandiol	0.43 ± 0.02	1.38 ± 0.04	a & b
39	45.69	1922	1925	(*E*,*E*)-Farnesyl acetone	0.16 ± 0.01	---	a & b
40	40.79	2045	2005	Isochiapin B	0.56 ± 0.02	1.03 ± 0.05	a & b
**Sesquiterpenes Hydrocarbons**							
41	19.26	1351	1351	*α*-Cubebene	2.19 ± 0.06	---	a & b
42	20.48	1376	1378	*α*-Copaene	0.84 ± 0.02	---	a & b
43	21.26	1409	1409	*α*-Cedrene	0.44 ± 0.03	---	a & b
44	21.71	1410	1412	*α*-Gurjunene	0.32 ± 0.01	---	a & b
45	22.29	1418	1418	*trans*-Caryophyllene	1.44 ± 0.07	3.07 ± 0.06	a & b
46	22.78	1429	1429	*cis*-Thujopsene	0.61 ± 0.03	0.83 ± 0.02	a & b
47	22.96	1462	1460	*α*-Humulene	0.26 ± 0.02	---	a & b
48	23.55	1480	1483	*α*-Muurolene	0.15 ± 0.01	---	a & b
49	23.81	1484	1486	Germacrene-D	0.19 ± 0.01	---	a & b
50	23.95	1496	1489	Aromadendrene	0.43 ± 0.02	---	a & b
51	24.64	1493	1496	*β*-Muurolene	0.56 ± 0.02	---	a & b
52	25.16	1500	1502	Bicyclogermacrene	---	4.37 ± 0.08	a & b
53	25.22	1517	1515	*α*-Selinene	0.95 ± 0.04	---	a & b
54	25.63	1521	1524	*cis*-Calamenene	0.74 ± 0.03	---	a & b
55	26.22	1532	1533	*γ*-Cadinene	1.86 ± 0.07	---	a & b
56	26.39	1537	1537	*α*-Cadinene	3.31 ± 0.06	---	a & b
57	26.56	1555	1557	Junipene	0.52 ± 0.03	---	a & b
58	27.45	1548	1546	*α*-Calacorene	0.16 ± 0.01	---	a & b
**Oxygenated Diterpenes**							
59	44.31	1942	1944	Isophytol	0.25 ± 0.01	2.66 ± 0.08	a & b
60	46.99	1949	1950	Phytol	8.73 ± 0.09	38.02 ± 0.13	a & b
61	47.95	2201	2203	*trans*-Geranyl geraniol	0.80 ± 0.02	3.24 ± 0.08	a & b
**Diterpenes Hydrocarbons**							
62	44.81	2064	2062	Kaur-16-ene	0.39 ± 0.03	0.90 ± 0.02	a & b
**Oxygenated Hydrocarbons**							
63	35.57	1635	1636	1-Heptatriacontanol	0.47 ± 0.03	---	a & b
64	41.35	1754	1756	Hexyl cinnamic aldehyde	0.91 ± 0.04	---	a & b
65	46.78	1927	1927	Methyl palmitate	2.86 ± 0.07	2.70 ± 0.05	a & b
66	47.23	2108	2109	Linoleic acid, methyl ester	---	0.72 ± 0.03	a & b
67	47.69	2128	2128	Methyl stearate	---	0.98 ± 0.04	a & b
68	47.78	2144	2145	*Z*-7-Hexadecenal	0.49 ± 0.03	2.10 ± 0.06	a & b
69	48.57	2152	2152	Linoleic acid	0.15 ± 0.01	6.36 ± 0.07	a & b
70	54.87	2161	2161	Oleic Acid	0.59 ± 0.02	3.51 ± 0.09	a & b
**Non-oxygenated Hydrocarbons**							
71	32.55	1900	1901	*n*-Nonadecane	---	0.45 ± 0.01	a & b
72	39.99	2000	2000	*n*-Eicosane	---	0.67 ± 0.02	a & b
73	43.24	2100	2101	*n*-Heneicosane	---	1.30 ± 0.05	a & b
74	49.33	2200	2200	*n*-Docosane	---	6.86 ± 0.11	a & b
75	52.45	2300	2303	*n*-Tricosane	0.84 ± 0.03	---	a & b
76	57.85	2500	2502	*n*-Pentacosane	0.76 ± 0.02	6.31 ± 0.07	a & b
**Aromatics**							
77	19.93	1355	1353	1,1,6-Trimethyl-1,2-dihydronaphthalene	0.37 ± 0.02	---	a & b
78	36.99	1438	1436	Bolandiol	0.17 ± 0.01	0.15 ± 0.01	a
79	38.42	1845	1843	Myristicin	6.13 ± 0.08	2.09 ± 0.05	a & b
80	40.73	1457	1457	Myristic acid	0.50 ± 0.02	---	a & b
81	44.51	2277	2478	Ethyl iso-allocholate	---	1.58 ± 0.06	a
**Carotenoid-derived compounds**							
82	17.41	1284	1283	Dihydroedulan II	0.16 ± 0.01	---	a & b
83	20.94	1351	1354	*α*-Damascenone	1.42 ± 0.03	0.71 ± 0.01	a & b
84	23.67	1426	1426	*trans*-*α*-Ionone	2.00 ± 0.05	2.91 ± 0.04	a & b
85	32.07	1473	1472	*α*-Iso methyl ionone	0.38 ± 0.03	0.38 ± 0.02	a & b
86	33.01	1518	1519	Methyl-*α*-ionone	0.19 ± 0.02	---	a & b
Total identified					99.11	98.80	

Rt: Retention time; KI_exp_: experimental Kovats retention index; KI_Lit_: Kovats retention index on DB-5 column with reference to *n*-alkanes; values are average ± SD. The identification of essential oil (EO) components was performed based on the (a) mass spectral data of compounds (MS) and (b) Kovats indices with those of Wiley spectral library collection and NIST (National Institute of Standards and Technology) library database.

**Table 2:** Antimicrobial activity of the essential oils (EOs) extracted from Saudi (SA) and Egyptian (Eg) ecospecies of *C. procera* at different concentrations.

Strains		EO Concentration (µg mL^−1^)						MIC ^b^ (µg mL^−1^)	Antibiotic (10 µg mL^−1^)
		2.50	1.25	0.625	0.312	0.156	0.078		
**Bacterial**									**Gentamycin**
*S.* *aureus*	SA	35.20 ± 0.60 ^a^	34.20 ± 0.95	33.20 ± 1.15	31.80 ± 1.00	30.10 ± 0.50	17.20 ± 0.80	15.75	36.00 ± 1.16
	Eg	23.00 ± 1.40	22.10 ± 0.77	20.20 ± 1.35	19.20 ± 1.30	16.00 ± 1.10	14.40 ± 0.80	15.75	
*S. pyogenes*	SA	22.00 ± 0.82	21.10 ± 0.85	18.40 ± 0.30	17.98 ± 0.89	16.00 ± 0.16	14.10 ± 1.20	20.58	30.00 ± 0.94
	Eg	22.80 ± 1.0	21.10 ± 1.43	19.00 ± 0.82	16.68 ± 0.80	14.90 ± 0.87	12.45 ± 0.90	15.79	
*S.* *epidermidis*	SA	20.00 ± 1.00	31.20 ± 0.90	28.10 ± 0.10	26.00 ± 0.65	24.60 ± 0.50	16.30 ± 0.98	15.75	34.00 ± 0.84
	Eg	33.00 ± 1.25	18.00 ± 1.00	17.20 ± 1.00	15.90 ± 1.00	14.68 ± 0.77	10.60 ± 0.35	15.81	
*S. typhi*	SA	37.00 ± 0.88	31.80 ± 0.65	29.20 ± 0.30	27.30 ± 0.25	25.70 ± 0.35	15.10 ± 0.73	20.58	37.00 ± 0.88
	Eg	32.30 ± 0.7	35.10 ± 0.85	33.78 ± 0.85	32.80 ± 0.70	30.90 ± 1.47	16.20 ± 1.20	15.75	
*E. coli*	SA	21.00 ± 1.10	9.10 ± 0.37	7.98 ± 0.10	6.97 ± 0.25	5.60 ± 0.19	6.20 ± 0.33	23.25	21.00 ± 1.10
	Eg	10.00 ± 0.52	20.20 ± 1.10	19.95 ± 1.07	17.20 ± 1.10	14.90 ± 0.56	8.50 ± 0.80	15.75	
*Shigella* spp.	SA	28.00 ± 1.10	37.00 ± 0.15	35.60 ± 0.40	33.60 ± 0.50	30.80 ± 0.35	17.50 ± 0.40	20.58	28.00 ± 0.60
	Eg	39.00 ± 0.7	26.20 ± 1.15	25.90 ± 1.10	25.00 ± 1.10	23.80 ± 0.64	11.00 ± 1.50	15.88	
*P. aeruginosa*	SA	31.00 ± 0.90	26.80 ± 0.95	25.20 ± 0.20	23.40 ± 0.25	30.10 ± 0.50	14.35 ± 0.70	31.25	31.00 ± 0.90
	Eg	28.00 ± 1.00	29.20 ± 0.90	27.00 ± 0.90	25.90 ± 0.90	23.50 ± 0.92	12.50 ± 1.30	23.25	
**Fungal**									**Amphotericin**
*T. shoenlenii*	SA	31.00 ± 0.90	29.20 ± 0.90	28.10 ± 1.10	25.90 ± 1.10	23.00 ± 1.10	15.00 ± 0.80	21.75	21.00 ± 1.10
	Eg	31.30 ± 1.15	30.10 ± 1.10	27.00 ± 0.90	25.90 ± 0.90	23.50 ± 0.96	12.50 ± 1.30	15.75	
*A. fumigatus*	SA	MI	MI	MI	MI	MI	9.00 ± 1.50	21.30	28.00 ± 0.60
	Eg	MI	MI	MI	MI	MI	35.00 ± 1.10	15.79	

^a^ Values are the average (*n* = 3) of the inhibition zone diameter (mm) ± standard deviation, ^b^ minimum inhibitory concentrations, MI: maximum inhibition (no growth at all), SA: Saudi, and Eg: Egypt.

## Supplementary Materials

The following are available online. Table S1: Two-way analysis of variance (ANOVA) of the allelopathic activity of the EOs from Egyptian and Saudi ecospecies of *C. procera* against the two weeds (*B. pilosa* and *D. aegyptium*), Table S2: Two-way analysis of variance (ANOVA) of antimicrobial activity of the EOs from Egyptian and Saudi ecospecies of *C. procera* against various bacterial and fungal strains.
