# Supplementary material for: Essential Oil of Calotropis procera: Comparative Chemical Profiles, Antimicrobial Activity, and Allelopathic Potential on Weeds
Source: Molecules. 2020 Nov 9;25(21):5203. doi: 10.3390/molecules25215203 (PMC7664932; doi:10.3390/molecules25215203)
Supplement: Supplementary file 1 [file molecules-25-05203-s001.pdf]

molecules

MDPI

Article

Essential Oil of *Calotropis procera*: Comparative Chemical Profiles, Antimicrobial Activity, and Allelopathic Potential on Weeds

Saud L. Al-Rowaily [1], Ahmed M. Abd-ElGawad [1,2,*], Abdulaziz M. Assaeed [1], Abdelbaset M. Elgamal [3], Abd El-Nasser G. El Gendy [4], Tarik A. Mohamed [5], Basharat A. Dar [1], Tahia K. Mohamed [6] and Abdelsamed I. Elshamy [6,*]

1 Plant Production Department, College of Food & Agriculture Sciences, King Saud University, P.O. Box 2460 Riyadh 11451, Saudi Arabia; srowaily@ksu.edu.sa (S.L.A.-R.); assaeed@ksu.edu.sa (A.M.A.), baseratali@gmail.com (B.A.D.)
2 Department of Botany, Faculty of Science, Mansoura University, Mansoura 35516, Egypt
3 Department of chemistry of Microbial and Natural Products, 33 El-Bohouth St., Dokki, Giza 12622, Egypt, algamalgene@yahoo.com
4 Medicinal and Aromatic Plants Research Department, National Research Centre, 33 El Bohouth St., Dokki, Giza 12622, Egypt; aggundy_5@yahoo.com
5 Chemistry of Medicinal Plants Department, National Research Centre, 33 El-Bohouth St., Dokki, Giza 12622, Egypt; tk.mohamed@nrc.sci.eg
6 Department of Natural Compounds Chemistry, National Research Centre, 33 El Bohouth St., Dokki, Giza, 12622, Egypt; ta.mourad@nrc.sci.eg
* Correspondence: aibrahim2@ksu.edu.sa (A.M.A-E.); ai.el-shamy@nrc.sci.eg (A.I.E); Tel.: +966-562680864 (A.M.A.-E.), Tel.: +201005525108 (A.I.E.)

Academic Editor: María Amparo Blázquez
Received: 13 October 2020; Accepted: 6 November 2020; Published: 9 November 2020

Supplementary Materials

Table 1. Two-way analysis of variance (ANOVA) of the allelopathic activity of the EOs from Egyptian and Saudi ecospecies of *C. procera* against the two weeds (*B. pilosa* and *D. aegyptium*).

Microbe	Effect	SS	df	MS	*F*	*P*
		B. pilosa				
Germination	Ecospecies (S)	2430	1	2430	108	< 0.001 ***
	Conc. (C)	33586.67	4	8396.67	373.19	< 0.001 ***
	S × C	2320	4	580	25.78	< 0.001 ***
Root gorwth	Ecospecies (S)	443.54	1	443.54	217.80	
	Conc. (C)	39233.66	4	9808.41	4816.47	
	S × C	157.10	4	39.28	19.29	
Root gorwth	Ecospecies (S)	721.92	1	721.92	51.72	< 0.001 ***
	Conc. (C)	36439.99	4	9110.00	652.72	< 0.001 ***
	S × C	539.98	4	134.99	9.67	0.002 ***
		D. aegyptium				
Germination	Ecospecies (S)	3796.88	1	3796.88	208.29	< 0.001 ***
	Conc. (C)	19700.52	4	4925.13	270.18	< 0.001 ***
	S × C	1085.94	4	271.48	14.89	< 0.001 ***
Root gorwth	Ecospecies (S)	5618.38	1	5618.38	266.62	< 0.001 ***
	Conc. (C)	19625.54	4	906.39	232.83	< 0.001 ***
	S × C	3836.06	4	959.02	45.51	< 0.001 ***
Root gorwth	Ecospecies (S)	9474.00	1	9474.00	538.90	< 0.001 ***
	Conc. (C)	14624.79	4	3656.20	207.97	< 0.001 ***
	S × C	3906.81	4	976.70	55.56	< 0.001 ***

Notes: Tested effects included ecospecies and concentration. For each tested effect, sum of squares (SS), degrees of freedom (df) mean squares (MS) and Duncan's test results (*F* and associated *s* value [significant values in boldface type]) are shown. Significance level fixed at *p*-values < 0.05. ***: significant at $p \leq 0.001$, ^{ns}: non-significant.

Table 2. Two-way analysis of variance (ANOVA) of antimicrobial activity of the EOs from Egyptian and Saudi ecospecies of *C. procera* against various bacterial fungal strains.

Microbe	Effect	SS	df	MS	*F*	*P*
			Bacterial strains			
S. aureus	Ecospecies (S)	979.90	1	979.90	781.08	< 0.001 ***
	Conc. (C)	586.97	5	117.39	93.58	< 0.001 ***
	S × C	204.23	5	40.85	32.56	< 0.001 ***
S. pyogenes	Ecospecies (S)	0.68	1	0.68	1.09	0.306 ns
	Conc. (C)	786.70	5	157.34	254.99	< 0.001 ***
	S × C	3.83	5	0.77	1.24	0.320 ns
S. epidermidis	Ecospecies (S)	826.18	1	826.18	1030.50	< 0.001 ***
	Conc. (C)	802.40	5	160.48	200.17	< 0.001 ***
	S × C	179.92	5	35.98	44.88	< 0.001 ***
S. typhi	Ecospecies (S)	134.48	1	134.48	298.89	< 0.001 ***
	Conc. (C)	313.90	5	62.78	139.53	< 0.001 ***
	S × C	29.32	5	5.86	13.03	< 0.001 ***
E. coli	Ecospecies (S)	710.49	1	710.49	951.30	< 0.001 ***
	Conc. (C)	403.16	5	80.63	107.96	< 0.001 ***
	S × C	148.60	5	29.72	39.79	< 0.001 ***
Shigella spp.	Ecospecies (S)	468.72	1	468.72	147.69	< 0.001 ***
	Conc. (C)	150.00	5	30.00	9.45	< 0.001 ***
	S × C	119.70	5	23.94	7.54	< 0.001 ***
P. aeruginosa	Ecospecies (S)	33.64	1	33.64	55.65	< 0.001 ***
	Conc. (C)	334.19	5	66.84	110.58	< 0.001 ***
	S × C	7.51	5	1.50	2.48	0.060 ns
			Fungal strains			
T. shoenlenii	Ecospecies (S)	135829.10	1	135829.10	0.94	0.3412 ns
	Conc. (C)	700372.45	5	140074.49	0.97	0.4543 ns
	S × C	729031.79	5	145806.36	1.01	0.4321 ns
A. fumigatus	Ecospecies (S)	33063.36	1	33063.36	9337.00	< 0.001 ***
	Conc. (C)	5761.14	5	1152.23	325.39	< 0.001 ***
	S × C	2081.14	5	416.23	117.54	< 0.001 ***

Notes: Tested effects included ecospecies and concentration. For each tested effect, sum of squares (SS), degrees of freedom (df) mean squares (MS) and Duncan's test results (*F* and associated *s* value [significant values in boldface type]) are shown. Significance level fixed at *p*-values < 0.05. ***: significant at $p \leq 0.001$, ns: non-significant.
